# The power of citation: giving credit where credit is due

**DOI:** 10.1242/dmm.052886

**Published:** 2026-03-05

**Authors:** Kirsty Hooper, Dina Mikimoto, Rachel Hackett, E. Elizabeth Patton

**Affiliations:** ^1^ The Company of Biologists, Bidder Building, Station Road, Histon, Cambridge CB24 9LF, UK; ^2^ MRC Human Genetics Unit and CRUK Scotland Centre and Edinburgh Cancer Research, Institute of Genetics and Cancer, Western General Campus, The University of Edinburgh, Edinburgh EH4 2XU, UK

## Abstract

**Summary:** Citations hold immense power in shaping the history of a field, the success of careers and the viability of journals. Accurate and considerate citation of the literature is, therefore, vital.

**Figure:**
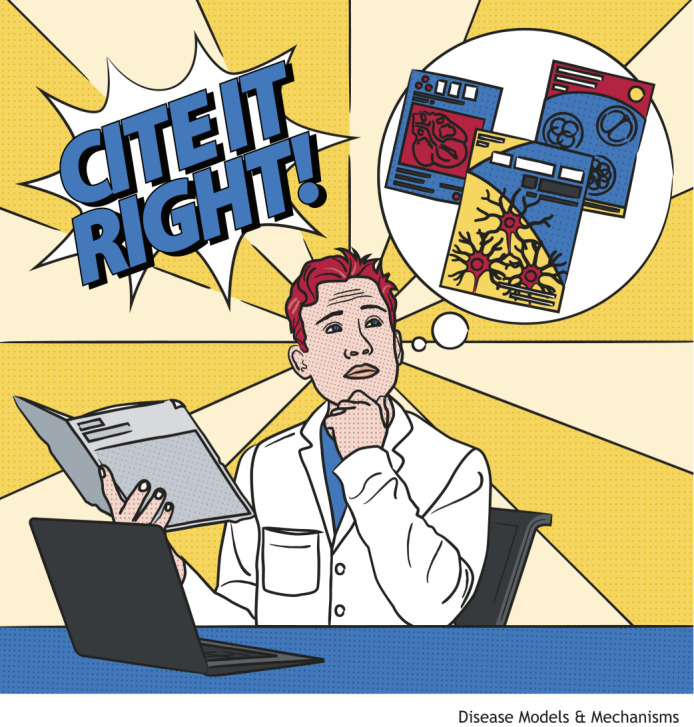
This image is by neilsmithillustration.co.uk and published under the CC-BY 4.0 license for this article.

Publishing a research paper is a powerful act (Briscoe and Brown, 2024). In addition to sharing new scientific results and conclusions with the community, publishing a paper requires citing the work of others to put the new results in the context of the field. Compared with the effort required to generate new scientific knowledge, this can seem like a minor part of the publication process. But, in fact, citations have the power to shape the history of the field, and can shine a light on both the overall direction of a field and, importantly, the gaps in understanding. Additionally, citations are often used as a metric for the success of careers and the viability of journals.

As part of our ongoing effort to improve and reflect upon how citations are applied in Disease Models & Mechanisms (DMM) papers, over the course of the past year, we held a series of individual conversations with members of our community, asking the question “How do you choose which papers to cite?”. The responses gave a fascinating insight into how laboratories from around the world put together manuscripts and raised some critical issues of concern.

## Getting right to the source

A major theme that emerged from our discussions was the value of citing the primary literature and its impact on shaping the field. Everyone agreed that it is critical to cite the primary source for specific statements, as outlined in the Declaration on Research Assessment (DORA)’s guidelines. But how does this change when there are multiple contributions to consider and a complex history of research that has built towards a discovery? For many scientific findings, it might simply be impractical to catalogue all the important contributions in an in-text citation, even if a journal does not limit the numbers of references, as is the case at The Company of Biologists’ journals (Box 1). Owing to these practical constraints, the ‘history’ of a discovery can end up being credited to a small cluster of the most prominent scientists in the field, or even the most prominent papers in a Google search, whether their papers were the most important for the discovery or not. Here, discussions with our community became especially interesting as we assessed the importance of Review articles in this context. Citing a high-quality Review alongside a mindful selection of primary literature can help ensure that readers are pointed towards a comprehensive account of a scientific discovery or field. Ideally, a great Review can present established facts in a balanced manner without overly selective citation of primary literature. This, therefore, also dissuades ‘citation bias’, which is the preferential citation of work that supports one's research and/or the exclusion of papers that contradict the hypothesis or findings (Gøtzsche, 2022).

In our discussions, it was emphasised that Reviews and Review-type articles can be especially beneficial to help researchers and students make sense of large, complex bodies of scientific information; provide an important foundation for scientific inspiration; identify gaps and unanswered questions; help evaluate the quality of evidence; and support teaching and learning. Review articles require deep knowledge and insight from people working in the field, so it is important to acknowledge their intellectual contribution to the progression of science. No process is perfect, and we acknowledge that each Review piece is written from a specific viewpoint regarding the literature, but citation bias can still occur within Review articles and can also arise when authors select which Reviews to cite, which can perhaps be swayed by the perceived impact of the journal in which it is published rather than the content and rigor of the article. Our Reviews Editors are trained to uphold scientific rigour and publishing integrity in our Reviews, and a major part of this is ensuring that they provide an unbiased account of the literature. This is why DMM continues to invest so much in our fully Open Access Review-type content – despite it not generating direct revenue for the journal.

Systematic reviews or meta-analyses, by nature, can be very unbiased sources, as studies included in these analyses are selected using predetermined criteria and not simply by human judgement (van Rhijn-Brouwer et al., 2024). Although they do not always foster critical or forward-looking discussion or address scientific nuance in findings like a classic review would, they can help to answer very specific research questions where a wide breadth of similar studies have been conducted. Therefore, these articles can act as reliable catalogues of scientific discovery that can be cited to help record the progress of a field.

Box 1. Credit where credit is due at The Company of Biologists DMM is part of a family of journals published by The Company of Biologists. Together, we continue to assess citation practices in our journals and how we can support authors to give credit where credit is due. Below, we outline our current activities in this area:

(1) We are signatories of the Declaration on Research Assessment (DORA) and follow their policies closely.

(2) We do not limit the number of references in an article.

(3) We encourage our authors to cite a fair and balanced account of the literature.

(4) Reviewers are encouraged to assess the references in an article, and we facilitate discussion between reviewers for a fair and thorough review process.

(5) Authors can include an Equity, Diversity and Inclusion statement in their articles, which can include citation demographics.

(6) Our journals have citation and inclusive citation policies in place, e.g. https://journals.biologists.com/dmm/pages/journal-policies#citation.

## Citation currency

A second theme that emerged was the impact of citations upon individuals. Citations are a currency in academia that can influence the perceived impact and quality of someone's work. With an increasingly competitive job market and funding landscape, researchers are under pressure to publish high-quality papers fast. DORA encourages hiring, promotion and funding decisions to be made based on the merit of the research itself rather than simply on the impact factor (IF) of the journal it is published in. This does mean that the citations an individual paper accrues remain very important to an individual's career progression. We, therefore, hold power when we cite other people's work, and we can truly support fellow scientists by recognising their high-quality and relevant science through the act of citing it. Properly recognising an individual's contribution to research fosters integrity, trust and career development (see UK Research Integrity Office's resources).

Unfortunately, ‘citation inequality’, a subtle variation of citation bias that may even be done subconsciously, is increasing in prevalence (Nielsen and Andersen, 2021), and the impact on individual researchers and the field can be substantial. Citation inequality is where a privileged sub-section of the literature is cited for reasons other than scientific merit. This can be based on the reputation of the authors, such as their seniority or authority in the field, or the prestige of their institute (Nielsen and Andersen, 2021), either because it is assumed that their research is the most important or to try to accrue favour with scientists who have a lot of influence in their field. This then introduces more insidious bias, such as geographical, gender or ethnicity bias. Articles written by authors based in countries that were traditionally more active in science are cited disproportionately more than articles from other countries, despite these countries significantly increasing their scientific output (Gomez et al., 2022; Mattiazzi and Vila-Petroff, 2024). Articles with women as the first and/or last author are cited disproportionately less than articles written by men across scientific disciplines (Chatterjee and Werner, 2021; Dworkin et al., 2020; Taube et al., 2025). Similarly, studies have found that there is an imbalance in citations for scientific articles written by non-White first and/or last authors (Chakravartty et al., 2018; Taube et al., 2025). Fewer studies have been done on ethnicity-based inequities in citations owing to a lack of robust author information (Kozlowski et al., 2022), but this is something that the joint commitment for action on inclusion and diversity in publishing is working to address. To ensure that we do not perpetuate disparity and inequality in academia (Sadler, 2023), we must ensure that our overview of the literature is based on merit and is accurate.

## Under pressure

A final area of concern that was raised by our community was the perceived pressure to cite ‘high-impact’ papers from ‘big-name’ journals at the start of the Introduction section to demonstrate that one's work belongs within this set of high IF journals. Selecting which articles to cite based on the IF or reputation of the journals in which they are published, rather than on the actual scientific content, perpetuates an unhealthy focus on IFs and is also against the values of appropriate credit practices that aim to ensure that all individuals who make substantive intellectual contributions to research are recognised. Even more worryingly, some of our community felt that Editors can imply that there is an expectation to cite the journal that the authors are submitting to right at the beginning of the Introduction to signal to the Editor that they will cite papers from their journals. ‘Journal self-citation’ is a common practice reflecting a field's interconnectedness, but it becomes inappropriate ‘citation manipulation’ when there is perceived pressure to artificially inflate the journal's IF. Coercive citation can also manifest as Editors and peer reviewers request the unnecessary citation of papers from their own journals or laboratories. Of course, this is distinct from a reviewer requesting citation of research that is very relevant to the topic and would be inaccurate to omit.

Connected to this is the emergence of papers that have very large author lists (more than 50) and garner high numbers of citations, perhaps, in part, owing to self-citations (Jaksic et al., 2023). It has been argued that this practice will inflate the citations for such a paper beyond its actual impact and does not always reflect the quality of the research. At The Company of Biologists, we have strict author contribution policies to ensure that all individuals listed as authors have made a significant contribution to the article, which dissuades this practice.

## Citation checkpoints

To increase awareness around the issues raised here and encourage more inclusivity, some journals invite authors to include citation diversity statements, in which they outline the demographics of the authors they have cited in their paper (Editorial, 2023; Ray et al., 2024; Zurn et al., 2020). At The Company of Biologists’ journals, authors are invited to include an Equity, Diversity and Inclusion statement for their article, in which they are welcome to include this information. Including these demographics should be done in a responsible manner. It is intended to highlight areas of the authors’ unconscious bias and encourage self-assessment, which may lead to the citation of more diverse authors, but only if their research is of relevance. It is not intended to encourage citation manipulation simply to ‘improve’ citation demographics. Furthermore, the responsibility of inclusive citation cannot solely be placed on authors. Another important checkpoint for citations is the peer-review process. DMM reviewers are asked to consider whether an article is adequately and appropriately referenced, and often provide helpful suggestions of papers that are highly relevant to the article being reviewed. As peer reviewers often assess the articles from a fresh perspective, they can broaden the authors’ awareness of the literature, which can even alter how the data are interpreted.

As the scientific publishing landscape continues to expand, it is becoming ever more challenging for researchers to meaningfully engage with all the literature in a given field. This is why artificial intelligence (AI) and large language models (LLMs) are being harnessed to retrieve and summarise relevant literature. However, limitations remain, most notably that LLMs often summarise the top fraction of articles, which narrows the focus of a field. Furthermore, current AI systems remain poor at assessing the appropriateness and credibility of sources, so human judgement and knowledge is still crucial to properly assess articles and enable accurate referencing. Despite these limitations, these tools could act as an important citation checkpoint to produce citation diversity statements, and, as they improve, we can hopefully become more reliant on them to highlight any important studies that have been missed.

## Conclusions

As a fully Open Access journal, DMM strives to communicate high-quality basic and translational research in disease biology to a broad audience of scientists, clinicians and patients. A key factor in this communication is to set the scientific scene by presenting an accurate and balanced account of the field. With guidance and insights from our community, DMM is optimising citation practice in our journal (Box 1). We encourage all our authors to be mindful when citing the literature (see image). Similar to the importance of selecting an ethical publisher (Briscoe and Brown, 2024), authors need to carefully consider the power they hold when citing the literature.
